# A Testability Strategy Optimization Method Under Multi-Valued Dependency Condition Based on Deep Reinforcement Learning

**DOI:** 10.3390/e28070733

**Published:** 2026-06-28

**Authors:** Chao Zhang, Yufei Zhang, Feng Wang, Xiaoxu Su, Zhijie Dong, Linlin Zuo

**Affiliations:** 1 Department of Integrated Technology and Control Engineering, School of Aeronautics, Northwestern Polytechnical University, Xi’an 710072, China; nendneke@mail.nwpu.edu.cn; 2 National Key Laboratory of Aircraft Configuration Design, Xi’an 710072, China; 3 Unmanned System Research Institute, Northwestern Polytechnical University, Xi’an 710072, China; fenfen@mail.nwpu.edu.cn; 4 The 6th Research Institute of China Electronics Corporation, Beijing 102209, China; suxiaoxu@ncse.com.cn (X.S.); dongzhj@ncse.com.cn (Z.D.); hhhlpbo@sohu.com (L.Z.)

**Keywords:** multi-valued dependency matrix, testability strategy optimization, deep reinforcement learning, deep Q-network

## Abstract

The multi-valued dependency matrix (MVD matrix) is an important testability modeling approach, which can deliver more comprehensive testability information than the traditional dependency matrix (D-matrix). However, existing testability strategy optimization algorithms perform poorly in handling the MVD matrix, and the high-dimensional MVD matrix further aggravates these limitations as system complexity increases. To address these problems, a novel testability strategy optimization method under multi-valued dependency conditions based on deep reinforcement learning (DRL) is proposed. Firstly, the sets of elements and two reward functions to minimize test sequence length and test cost are established from the MVD matrix. Subsequently, the algorithm for selecting test points based on Deep Q-Network (DQN) is proposed. The DQN parameters are updated to fit the Q-value of test points. Thirdly, Double DQN (DDQN) and the prioritized experience replay (PER) mechanism are introduced to address the overestimation problem and sample redundancy problem, respectively, in high-dimensional matrix environments. The experimental results show that the testability strategy generated by this method can isolate all faults with fewer steps or at a lower cost. In a high-dimensional matrix environment, it can reduce test costs compared with the other heuristic algorithms while maintaining a good level of stability.

## 1. Introduction

Testability is an inherent characteristic of a product, which refers to the ability to conveniently, quickly, and accurately determine its state and isolate internal faults under specified conditions and within a given time frame. The main purpose of testability is to shorten the time or cost for fault isolation, thereby ensuring the reliability and maintainability of the product [1,2,3,4].

In testability modeling, the dependency matrix (D-matrix) is the most widely used and the most intuitively described method [5,6,7,8]. The traditional single-value D-matrix uses binary logical values, with two Boolean logics respectively indicating whether the test point can detect the fault [9,10,11]. For complex systems, due to the extremely large number of faults and test points, the testing efficiency of the traditional D-matrix is often very low [12]. A dependency matrix with multiple test values is referred to as a multi-valued dependency matrix (MVD matrix) [13], and it reflects the relationship between faults and tests in a multi-valued form. Currently, testability modeling based on the MVD matrix method has been widely applied in various fields. Huang et al. [14] classified the relationship between faults and test points into three categories, thereby improving the isolation efficiency of the MVD matrix. Su et al. [15] conducted MVD matrix-based fault testability analysis for engine systems. Zhu et al. [16] proposed a new method for constructing the four-valued D-matrix to conduct testability modeling for the fuel metering system of the aero-engine. The use of an MVD matrix enables the acquisition of richer test result information and improves the test efficiency of the model.

The primary role of the D-matrix is to assist testers in formulating an optimal testability strategy, thereby enabling the corresponding test point to isolate all faults with fewer steps or lower costs [17,18]. Currently, many scholars have conducted relevant studies on this issue [19,20,21]. For instance, Liang et al. [22] proposed an optimal testability strategy method based on the partially observable Markov decision process (POMDP) to address the problem of unreliable test strategy. Cui et al. [23] introduced hash functions to convert the dependency matrix into key-value pairs, and utilized the binary search algorithm to locate the required fault modes. Zhang et al. [24] combined the advantages of the classic algorithms’ information gain and fault detection weight index to improve the optimization efficiency. Wang et al. [25] solved the complex system fault test strategy problem using swarm intelligence algorithms, resulting in an 8–10 times improvement in testability performance.

However, these methods are only applicable to the traditional binary D-matrix and have not been extended to the MVD matrix level. When dealing with multi-valued testing, many of these methods either become inapplicable or yield poor results. Currently, there are relatively few studies on optimizing testability strategy based on the MVD matrix, and the existing research mainly focuses on the application of heuristic algorithms [15,16]. Dandan et al. [26] proposed a multi-value testability strategy optimization method based on information entropy, but this greedy algorithm is prone to getting stuck in a local optimal solution. Tian et al. [27] introduced the swarm optimization algorithm into the solution of the multi-valued system test strategy, but the algorithm is limited by its computational complexity. As modern equipment becomes larger and more complex, the dimensionality of the MVD matrix increases sharply. Consequently, the inherent limitations of existing optimization methods become more pronounced. This leads to an exponential growth in computational costs when existing algorithms are applied to complex systems, which not only makes it difficult to obtain the optimal solution, but also results in excessively long operation time.

Deep reinforcement learning (DRL) based on the Markov decision model is an integrated technology combining deep learning and reinforcement learning, and its core idea is to enable an agent to independently explore the optimal strategy through “trial-and-error learning” in a complex environment [28,29]. Moreover, it can also utilize the deep neural network to perform value fitting, thereby addressing the combinatorial explosion problem in the state space [30]. Currently, DRL has been widely applied in fields such as target recognition [31], path planning [32], and intelligent decision-making [33].

The core of the testability strategy solution based on the MVD matrix lies in the decision-making and selection process of test points, which can be regarded as a Markov decision process. DRL can learn the optimal action plan more quickly, which essentially means exploring the optimal testability strategy. When facing the high-dimensional MVD matrix, DRL can significantly enhance the efficiency and basic accuracy of the testability strategy optimization process compared with traditional algorithms, and can also provide a more reliable test strategy.

Therefore, this paper proposes a novel testability strategy optimization method based on the idea of deep reinforcement learning. Taking the sampling circuit and the multi-functional integrated radio frequency system (MIRFS) as the research objects, respectively, the feasibility of this method is verified. Firstly, the Markov decision model is established based on different optimization goals of the testability strategy to determine the reward direction for the intelligent agent. Secondly, a Deep Q-Network (DQN) is proposed to select the optimal test point under different fault isolation states. Thirdly, Double DQN (DDQN) and prioritized experience replay (PER) mechanisms are introduced, respectively. The model trained can output the optimal test strategy and diagnostic tree. The main contributions of this paper are as follows:

(1) The Markov decision model for solving the testability strategy is established. Firstly, the intelligent agent, state set, action set and state transition probability are defined from the MVD matrix. Then, the reward functions for testability strategy optimization are established, and the test sequence length or test cost is linked to the reward functions. Based on the idea of accumulating the maximum reward in a Markov decision process, the agent will strive to minimize the length of the test sequence or the test cost while isolating all faults.
(2) A novel test point optimization algorithm based on the DQN is proposed. A deep neural network is used to approximate the Q-value functions, and the selection of the test point will be based on the neural network. Experience samples are obtained through the MVD matrix, and the parameters of the Q-network are moderately updated through sample learning based on the Markov decision model. The update of parameters enables the fitting of the Q-value of test points to converge towards the shortest test sequence or the lowest test cost.
(3) DDQN and PER mechanisms are introduced to mitigate overestimation and redundant experience samples problems, respectively. As the MVD matrix structure continues to expand, the overestimation problem of the DQN will be severe. Meanwhile, the experience samples will also become complex and redundant, which leads to a decrease in the training efficiency of the model. Therefore, the DDQN and the PER mechanism are respectively introduced to construct the Prioritized Experience Double Deep Q-Network (PE-DDQN) algorithm model. It can alleviate the overestimation problem and make the algorithm learn the optimal action plan more effectively.

The experimental results show that the testability strategy optimization method proposed in this paper can effectively isolate all faults with fewer steps or at a lower cost. In a contrast experiment conducted in a high-dimensional matrix environment, it can reduce test costs compared with the other heuristic algorithms while maintaining a good level of stability. For high-dimensional MVD matrices of complex systems, this method has strong engineering application prospects and promotion value.

The core contribution of this study lies in the development of a novel framework based on the existing DRL algorithms, which is structurally tailored for testability strategy optimization under multi-valued dependency conditions. By strategically integrating mature DRL techniques into the generation of testability strategies, this work significantly enhances the applicability of modern AI algorithms in solving practical engineering testing problems.

## 2. Problem Description and Model Construction

### 2.1. Problem Description

The test strategy optimization problem is a process aimed at designing a series of tests with a specific strategy based on a predefined quintuple. The core objective is to effectively identify and isolate the potential fault state set $F=\{f_{1},f_{2},\ldots ,f_{m}\}$ using a strategically designed sequence of $T=\{t_{1},t_{2},\ldots ,t_{k}\}$.

#### 2.1.1. MVD-Matrix

The MVD matrix is an extended form of the D-matrix for the analog signal test, which represents the matrix element values as the number of different fault fuzzy groups according to the effective value of test signals [34,35]. Its row vectors represent the test values of a certain fault at multiple test points, and a fault is determined when the actual test values completely match the corresponding row vector. The MVD matrix has an element value range of 0 to k-1, which is shown as

(1) $$D=\left[\begin{array}{cccc} d_{11} & d_{12} & \ldots & d_{1n}\\ d_{21} & d_{22} & \ldots & d_{2n}\\ \vdots & \vdots & \vdots & \vdots \\ d_{m1} & d_{m2} & \ldots & d_{mn} \end{array}\right]$$

When constructing the MVD matrix, the following two sets need to be determined first:

(1) Fault Set

$F=\{f_{1},f_{2},\ldots ,f_{m}\}$ represents the set of possible failure modes that may occur in the entire system, where *m* denotes the total number of failure modes. Each failure $f_{i}$ represents an independent failure that may cause functional failure in the system.

(2) Test Set

$T=\{t_{1},t_{2},\ldots ,t_{k}\}$ represents the set of available test items in the system, where k denotes the number of test items. Each test $t_{j}$ is a tool used to detect or verify a certain fault state.

On this basis, the MVD matrix needs to be expanded with two additional parts:

(1) Prior Probability Set:

$P=\{p_{1},p_{2},\ldots ,p_{m}\}$ represents the prior probability associated with each fault $f_{i}$ in the fault state set, and the sum of these probabilities is 1.

(2) Cost Set:

$C=\{c_{1},c_{2}\ldots ,c_{n}\}$ represents the cost incurred by using the test point $t_{i}$, which includes the manpower, material resources, financial resources, and time consumed.

#### 2.1.2. Optimization of Testability Strategy

Isolating all faults rarely requires utilizing every test point. Due to inherent redundancy, selecting a targeted subset is usually sufficient. Meanwhile, the sequence order also has an impact on the isolation of faults. Under different sequence orders, the sequence of fault isolation and the speed of isolation will vary, which will affect the speed of troubleshooting when equipment fails. Furthermore, the testing plan can be dynamically adjusted based on the outcome of each test, which is the primary purpose of a testing strategy. The testability strategy can also be depicted in the form of a diagnostic tree.

For the optimization of the testability strategy, there are two specific directions:

(1) The test sequence that isolates all faults contains the fewest test points. The elements of the set $T_{f}$ of the fault isolation set F are the fewest, as shown in

(2) $$L=|T_{f}|.$$

This optimization direction focuses on the minimum identification steps for fault isolation. Based on the assumption of the homogeneity of test operation costs, it achieves rapid fault location by constructing the smallest independent test set. Essentially, it is the solution to the smallest complete test subset within the framework of combinatorial optimization, and is applicable to scenarios where test resources are homogeneous and the constraints are mainly time complexity.

(2) When all faults are isolated, the weighted test cost is minimized. The calculation formula for the test cost is shown as

(3) $$J=\sum_{i=1}^{m}\left(\sum_{j=1}^{\left|T_{f_{i}}\right|}c_{T_{f_{i}}[j]}\right)p(f_{i}),$$

where $p(f_{i})$ is the prior probability of fault i; $T_{f_{i}}$ is the test sequence used for isolating fault $f_{i}$; $\left|T_{f_{i}}\right|$ is the length of the test sequence; $c_{T_{f_{i}[j]}}$ is the cost of the j-th test in the test sequence list for isolating fault $f_{i}$. In order to make the expected test cost universal, this paper sets the test cost $c_{T_{f_{i}[j]}}$ as a dimensionless physical quantity.

This direction involves expected cost optimization under probability weighting. The cost is weighted by the prior probability of failure, and the heterogeneity costs of the testing operations are considered. Through Bayesian inference and dynamic programming, the statistical average cost of fault isolation is minimized, corresponding to scenarios such as the full life cycle maintenance of complex equipment and the allocation of high-cost testing resources.

Our goal is to minimize the test sequence length or the test cost while isolating all faults.

### 2.2. Model Construction

With the increase in fault types and test points, the dimensions of the MVD matrix model become extremely large, and traditional optimization methods perform poorly. The DRL optimization method is based on Markov decision processes (MDPs) [36], and it can find more efficient and accurate solutions, which provides a new approach for the test optimization of multi-valued systems. Fundamentally, the exact underlying fault in the system is hidden, which traditionally formulates the test strategy optimization problem as a POMDP. However, to efficiently apply DRL, we resolve this by utilizing the concept of information state. By tracking the current set of suspected faults based on past test outcomes, the next ambiguity state strictly depends only on the current ambiguity state and the selected test. Therefore, we reformulate the problem into a fully observable MDP over the information space. This provides a rigorous and consistent theoretical basis for our DRL framework. In this section, we establish an MDP model based on the MVD matrix and combine this model with the optimization strategy to form a complete framework for solving the testability strategy optimization problem.

#### 2.2.1. Set of Elements

(1) Agent:

The agent leverages accumulated experience to maximize rewards and learns from past suboptimal choices. In this framework, the agent’s actions correspond to the selection of test points, aiming to choose the optimal test point in any given state.

(2) State set

Assume the current system’s MVD matrix contains m fault modes and n test points, with multi-valued elements ranging from 0 to k−1. We strictly define the agent’s state as a three-dimensional tensor. The principal difficulty in applying reinforcement learning to high-dimensional MVD matrices lies in the definition of states: as the matrix grows, the state space becomes high-dimensional and only partially predictive of the eventual isolation reward, a challenge identified as central to deploying reinforcement learning beyond tractable laboratory conditions, where states are high-dimensional, continuous, and partially observable [37]. The three-channel state encoding adopted here is one attempt to expose the reward-relevant subset of state dimensions to the network.

The three channels extract data from different physical and logical dimensions of the environment:

Channel 1: static MVD matrix

This serves as the foundational base map of the environment, providing the network with the global static test response patterns.

(4) $$C_{i,j}^{\left(1\right)}=\frac{d_{i,j}}{k-1},$$

where $d_{i,j}$ represents the original matrix element, normalized by dividing by the maximum value k−1 to ensure a stable input distribution for the network.

Channel 2: dynamic fault row-mask

This channel reflects the real-time “survival status” and “fuzziness” of each fault, guiding the convolution kernels to focus on rows that have not yet been isolated.

(5) $$C_{i,j}^{\left(2\right)}=\left\{\begin{array}{cc} 0, & \mathrm{if}\;\mathrm{fault}\;f_{i}\;\mathrm{is}\;\mathrm{completely}\;\mathrm{isolated}\\ \frac{\left|g_{x}\right|}{m}, & \mathrm{if}\;\mathrm{fault}\;f_{i}\;\mathrm{remains}\;\mathrm{in}\;\mathrm{fuzzy}\;\mathrm{group}\;g_{x} \end{array}\right.,$$

where rows of isolated faults are zeroed out entirely. Faults still in a fuzzy group are assigned the relative size of that group. By encoding the dynamic survival status of the faults into this channel, the agent is provided with a fully observable belief state, perfectly aligning with the aforementioned MDP formulation.

Channel 3: action column-mask

This records the consumption of the test sequence, directly cutting off the feature transmission of executed actions within the 2D space.

(6) $$C_{i,j}^{\left(3\right)}=\left\{\begin{array}{cc} -1, & \mathrm{if}\;\mathrm{test}\;\mathrm{point}\;t_{j}\;\mathrm{has}\;\mathrm{been}\;\mathrm{executed}\\ 1, & \mathrm{if}\;\mathrm{test}\;\mathrm{point}\;t_{j}\;\mathrm{has}\;\mathrm{not}\;\mathrm{been}\;\mathrm{executed} \end{array}\right.,$$

where executed test columns are entirely set to −1. Paired with the subsequent activation function, this creates an excellent dead zone masking effect, preventing the network from evaluating already-taken actions.

(3) Action set

Action set represents the actions that the agent can take in each state. Actions are usually represented by the symbol A. In this model, the elements of the action set are defined as the execution of individual tests, and it can be expressed as

(7) $$A=T=\{t_{1},t_{2},\ldots ,t_{n}\},$$

where n represents the total number of test points in the MVD matrix.

(4) State transition probability

The state transition probability describes the probability of moving to another state after taking a specific action in a certain state. It is usually denoted by $P(s_{j+1}|s_{j},a_{j})$, which represents the probability of transitioning from state $s_{j}$ to state $s_{j+1}$ given action $a_{j}$.

Under the optimization goal of sequence length, we only need to consider the minimality of the test sequence. By default, each test will be conducted on all the fuzzy groups. Therefore, the state transition is directly defined by the changes in the fuzzy groups before and after the test, thereby forming a deterministic transition probability. In contrast, under the cost optimization objective, different branches can adopt different testing schemes. The environment simulates a single test response based on the faults’ prior probability distribution, directing the system to a specific fault subset. The model will form a unique subsequent state based on this subset of fuzzy groups. This MVD matrix-driven physical transition prevents the agent from falling into superposition states and ensures it learns the true environmental dynamics, indicating a probabilistic rather than fixed state transition mechanism.

**Figure 1:**
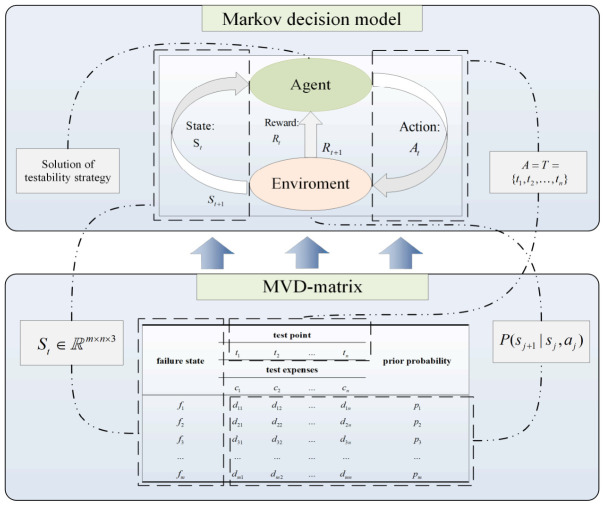
Model construction process.

The relationship diagram of the Markov model obtained from the MVD matrix is shown in Figure 1.

#### 2.2.2. Reward Function

For the Markov decision process, the goal of the agent is to accumulate the maximum reward. The reward function $R\left(s_{j},a_{j},s_{j+1}\right)$ represents the reward obtained when taking action $a_{j}$ and transitioning from state $s_{j}$ to state $s_{j+1}$. By executing an action, the agent will transition from one state to another. After executing an action in a certain state, the agent will receive a reward. Through continuous trial and error and iterative updates, eventually, the scores of each action in different states are obtained. Based on these score values, testability strategy decisions will be generated.

(1) The shortest test sequence

For the shortest test sequence, the classic constant step penalty method is adopted. By using dense negative feedback, the intelligent agent is forced to search for the shortest path. The basic idea is that when the agent isolates all the fault fuzzy groups, a positive reward is given; otherwise, a small negative reward will be given. This indicates that when the agent moves from state $s_{j}$ to state $s_{j+1}$ by taking action $a_{j}$, if the total number of completely isolated faults in state $s_{j+1}$ reaches the total number of faults, a positive reward is given, and a small negative reward is given in other states. At the same time, when the agent’s state reaches the above condition, it means that all fault isolation has been completed, and the exploration ends at this point. This reward mechanism ensures that the agent prioritizes ensuring that only the shortest test sequence can isolate all fault groups, thereby achieving optimality in the direction of the shortest test sequence. Its reward function is shown as

(8) $$R\left(s_{j},a_{j},s_{j+1}\right)=\left\{\begin{array}{cc} r_{pos}, & G_{total}=m\\ -\varepsilon , & other \end{array}\right.,$$

where $r_{p}$ represents a large positive terminal reward for complete fault isolation; ε is a strictly positive scalar representing a small fixed step penalty; and m represents the total number of faults. This mechanism ensures that the agent pays a consistent penalty −ε for every redundant or inefficient test, thereby driving the Q-learning process to prioritize the absolute shortest path to reach $r_{p}$.

(2) The minimum test cost

Regarding the minimum testing cost, since directly using negative reward costs would lead to inconsistent punishment scales and exploration failure, an innovative cost savings margin method is proposed, which can utilize a purely forward incentive without negative feedback to guide the network to converge. The rewards rules are as follows:

(1) For any given state transition, if the action $a_{j}$ fails to isolate any faults in the currently explored fuzzy group, no reward is given.

(2) When the current fuzzy group is completely isolated down to a single specific fault $f_{i}$, the current episodic exploration terminates. According to Equation (3), a terminal reward $R_{terminal}$ is given based on the cost savings margin. To calculate this, let $T_{seq}$ represent the sequence of tests executed by the agent in the current episode. The terminal reward is formulated as

(9) $$R_{terminal}=\eta \left(C_{total}-\sum_{t_{j}\in T_{seq}}c_{j}\right),$$

where $c_{j}$ is the cost associated with executing test $t_{j}$. As established in Section 2.1.2, both $c_{j}$ and $C_{total}$ are treated as dimensionless physical quantities to ensure universal applicability across different system parameters. The parameter η is a positive reward scaling coefficient.

Consequently, the step-by-step reward function $R_{t}$ is shown as

(10) $$R_{t}=\left\{\begin{array}{cc} R_{terminal}, & \mathrm{if}\;\mathrm{isolated}\;\mathrm{to}\;\mathrm{single}\;f_{i}\\ 0, & \mathrm{otherwise} \end{array}\right..$$

While standard pathfinding algorithms often rely on negative step penalties, this cost optimization model uniquely achieves convergence using a purely forward, non-negative incentive. Because the fault isolation process is framed as a strictly finite-horizon episodic task, an inverse-cost terminal payout is mathematically sufficient to guarantee policy convergence. By ensuring that taking fewer, cheaper tests results in a strictly larger $R_{terminal}$, the optimal policy naturally aligns with the minimum-cost sequence. Furthermore, the coefficient η acts as a crucial scaling parameter. Empirical tuning of subsequent experiments indicates that establishing η to map the terminal reward into a moderate numerical range (e.g., $10^{1}$ to $10^{2}$ as reflected in the stable Q-value convergence curves in Section 4) yields the highest training stability. This constraint prevents extreme magnitude variance in the target network, effectively mitigating gradient explosion or vanishing, and ensuring the smooth convergence of the PE-DDQN model while maintaining sufficient numerical resolution to distinguish subtle cost differences.

During the output process of the diagnostic tree, when optimizing the testing cost, the cost generated by isolating a fuzzy group through multiple tests is different. In this case, it is stipulated that each fuzzy group can use different testing methods. Therefore, for each unisolated fuzzy group, a recursive state traversal mechanism is adopted. Each fuzzy group constructs an independent set of state spaces and independently selects the optimal action to ensure that the weighted cost can be minimized.

## 3. The Proposed Testability Strategy Optimization Algorithm

**Figure 2:**
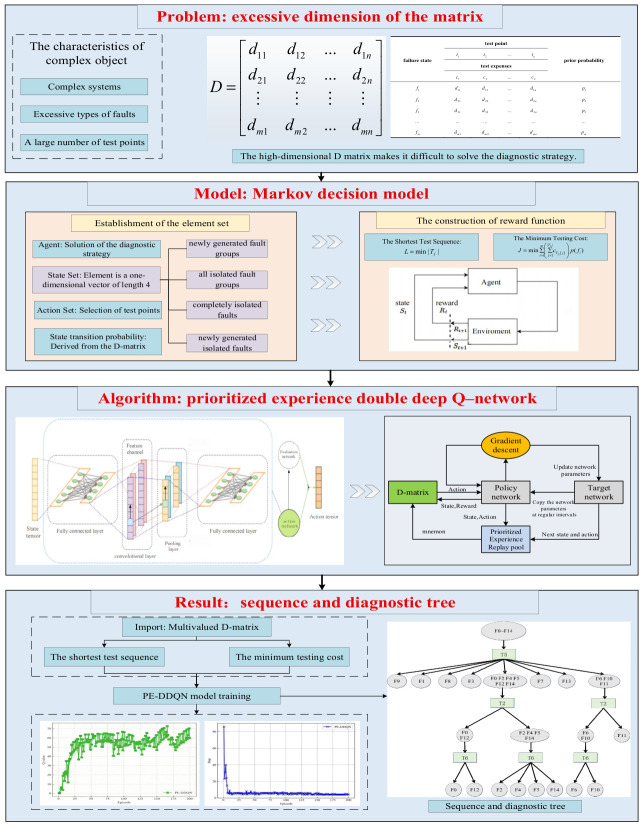
Testability strategy solution flowchart.

After constructing the element set and reward functions in the Markov decision model based on the MVD matrix, it is necessary to use an effective DRL algorithm to solve the Markov decision problem. This section proposes a solution algorithm based on the DQN. By continuously obtaining samples from the MVD matrix to train the DQN and updating its parameters, the network accurately estimates the Q-value of test points in each state, providing a basis for selecting test points. At the same time, the PER and DDQN are introduced to solve the overestimation problem and the problem of excessive sample size that occur during the model training process due to the high-dimensional MVD matrix. The DQN trained by the two proposed reward mechanisms can respectively generate test strategies and diagnostic trees for the two optimization goals. The flowchart of solving the testability strategy for the MVD matrix is shown in Figure 2.

### 3.1. The Test Point Optimization Strategy Based on DQN

During the test point selection process, the agent’s goal is to maximize the cumulative reward. Therefore, it needs to add the maximum reward that can be obtained in future states to the reward for achieving the current state. The core of the algorithm utilizes the Bellman equation as the value iteration update [38,39], which is shown as

(11) $$Q^{\mathrm{new}}(s,a)\leftarrow Q(s,a)+\alpha [r+\gamma \;\underset{a^{\prime }}{\max}Q(s^{\prime },a^{\prime })-Q(s,a)],$$

where $Q^{\mathrm{new}}(s,a)$ is the updated Q-value of the agent’s state s for action a; Q(s,a) is the Q-value of the intelligent agent’s state s and action a before the update; $Q(s^{\prime },a^{\prime })$ is the next state $s^{\prime }$ of the updated agent, along with the Q-value for each possible action; r is the reward obtained by the current action a interacting to transform into the next state $s^{\prime }$; a is the learning rate; γ is the discount factor.

**Figure 3:**
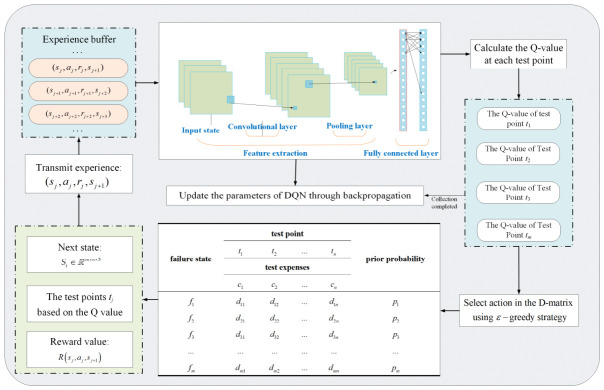
Q-network structure.

The DQN algorithm uses certain deep neural networks to approximate the Q-value functions based on Q-learning [40,41]. Figure 3 presents a reinforcement learning training framework for test point selection based on DQN. The environment is represented by the MVD matrix, which includes fault states, test points, detection capabilities, and costs. DQN employs a greedy strategy to obtain experiences from the MVD matrix and conducts model parameter training. After the DQN model is trained, the initial state is input into the trained DQN and continuously selects the test point with the highest score to generate a testability strategy. Based on this, the steps for building DQN to solve the testability strategy are as follows:

Step 1. Initialization of network and parameters

To stabilize the training process, the standard DQN algorithm utilizes two neural networks with identical structures but distinct parameter sets. Firstly, the DQN is initialized, including the policy network $Q_{1}$ and the target network $Q_{2}$. The network parameters are defined: let θ denote the parameters of the policy Q-network, which is continually updated at each step; let $\theta^{-}$ denote the parameters of the target Q-network, which is used to calculate the target value and is only updated periodically. The networks consist of convolutional layers, pooling layers, and fully connected layers, as shown in Figure 3. The networks take the three-channel state space vector as the input, and further capture the coupling relationship between the number of fault groups and the isolation efficiency through convolution-pooling operations. The fully connected layer is the last layer of the convolutional neural network, which is used to fuse and classify the features extracted by the convolutional neural network. In DQN, each element of the output vector corresponds to the Q-value estimate of an action. The Q-value estimates obtained by flattening the output of the previous layer of the fully connected layer are the basis for the agent to make action decisions.

During the DQN training process, the agent can adopt the ε -greedy strategy to select actions, thereby ensuring the comprehensiveness of sample coverage. In other words, the agent will select the action with the highest Q-value with a probability of 1−ε to exploit the learned policy, and explore other random actions with a probability of ε.

Step 2. Experience collection

The agent interacts with the MVD matrix by selecting actions based on the output of the fully connected layers of the policy network. After performing an action, the environment provides a reward and transitions to the next state. The agent stores this experience $\left(s_{j},a_{j},r_{j},s_{j+1}\right)$, including the current state, the executed action, the obtained reward, and the next state, in the buffer pool. If the buffer pool is full, new experiences will overwrite the earliest stored ones.

Step 3. Sampling and calculation of Q-value

A batch of experiences is randomly sampled from the buffer pool. Assume that the batch size is N. These samples are obtained through uniform random sampling from the replay buffer, and each sample contains a complete experience $\left(s_{j},a_{j},r_{j},s_{j+1}\right)$. For each sampled experience, the Q-value $y_{j}$ is calculated. If the next state $s_{j+1}$ is a terminal state, then $y_{j}=r_{j}$; otherwise, the Q-value calculation formula is

(12) $$y_{j}=r_{j}+\gamma \;\underset{a}{\max}Q_{2}\left(s_{j+1},a;\theta^{-}\right),$$

where $y_{j}$ is the target Q-value of the j-th sampling experience; $r_{j}$ is the immediate reward obtained by the agent when it transitions from state $s_{j}$ to state $s_{j+1}$ after taking action $a_{j}$ in the j-th sampling experience; γ is the discount factor; $Q_{2}(s_{j+1},a;\theta^{-})$ is the Q-value estimate for taking action a in state $s_{j+1}$, calculated through the target network; $\theta^{-}$ is the parameters of the target network.

Step 4. Loss calculation and backpropagation

For each sampled empirical sample, the current state $s_{j}$ is input into the Q-network to obtain the predicted Q-value corresponding to the executed action $a_{j}$.

The loss function adopts the mean square error (MSE), and its expression is shown as

(13) $$L(\theta )=\frac{1}{N}\sum_{j}{\left(y_{j}-Q_{1}(s_{j},a_{j};\theta )\right)}^{2}.$$

This loss function measures the difference between the predicted Q-value and the target Q-value. The training objective is to minimize this loss function, so that the Q-network can better approximate the target Q-value.

Based on the calculated loss, the parameters θ of the policy network are updated through the backpropagation algorithm. The backpropagation algorithm starts from the loss function and calculates the gradients along the hierarchical structure of the Q-network in reverse. The parameters of the network are updated according to the gradients and the learning rate. The gradient update of the parameters is shown as

(14) $$\theta \leftarrow \theta -lr\cdot \nabla_{\theta }L(\theta ),$$

where lr is the learning rate.

Step 5. Update of target network

After a certain number of steps, the target network needs to be checked and updated. If the update conditions are met, the parameters of the policy network are copied to the target network:

(15) $$\theta^{-}\leftarrow \theta .$$

The parameters of the target network will keep up with the update pace of the policy network and maintain the relative stability.

### 3.2. The Optimization of the DQN Model—PE-DDQN

As the system becomes increasingly complex, the high-dimensional MVD matrix exacerbates the overestimation issue in DQN caused by selecting the maximum Q-value during training and error accumulation. The large five-tuple matrix structure increases the diversity of experience samples, while traditional experience replay cannot prioritize important samples. To address these problems, we combine DDQN and PER to construct the PE-DDQN model to adapt to the high-dimensional MVD matrix environment.

As the system becomes increasingly complex, the high-dimensional MVD matrix exacerbates the overestimation issue in DQN caused by selecting the maximum Q-value during training and error accumulation. The large five-tuple matrix structure increases the diversity of experience samples, while traditional experience replay cannot prioritize important samples. To address these problems, we combine DDQN and PER to construct the PE-DDQN model to adapt to the high-dimensional MVD matrix environment. The overestimation and sample-redundancy problems noted above reflect a broader observation that, in high-dimensional and continuous settings, reinforcement learning must contend with sparse, noisy experience and with the tension between model-free and model-based updating; stable, reward-relevant state representations have been argued to be a prerequisite for reliable learning under these conditions [42]. In the present method, the three-channel encoding together with prioritized experience replay and the decoupled Double-DQN targets serve this role, suppressing the overestimation and sample-redundancy effects that the high-dimensional MVD matrix would otherwise amplify.

#### 3.2.1. DDQN

The overestimation problem exists in the aggressive Q-learning algorithm. DDQN is mainly designed to address the suboptimal strategy issue caused by the overestimation of action values in Q-learning. It separates action selection from action evaluation. When choosing the next action $a^{*}$, the $Q_{1}$ function is used for selection, which is shown in

(16) $$a^{*}=\arg\underset{a}{\max}Q_{1}(s_{j+1},a).$$

When calculating the target value, another function $Q_{2}$ is used to evaluate the value of this action. The formula for updating the target value becomes

(17) $$Q_{1}(s_{j},a_{j})\leftarrow Q_{1}(s_{j},a_{j})+\alpha [r_{j}+\gamma Q_{2}(s_{j+1},a^{*})-Q_{1}(s_{j},a_{j})].$$

This operation can reduce the overestimation phenomenon caused by always choosing the maximum estimate value. By regularly exchanging the parameters of $Q_{1}$ and $Q_{2}$, the two Q-networks can both participate in action selection and evaluation, ensuring fairness and accuracy.

#### 3.2.2. PER Mechanism

PER breaks the uniform sampling of traditional experience replay, and prioritizes learning important samples based on temporal difference error (TD-error). For an experience sample $\left(s_{j},a_{j},r_{j},s_{j+1}\right)$, the formula for calculating the TD-error is

(18) $$\delta =r_{j}+\gamma Q_{2}(s_{j+1},\arg\underset{a_{j+1}}{\max}Q_{1}(s_{j+1},a_{j+1},\theta );\theta^{-})-Q_{1}(s_{j},a_{j};\theta ).$$

The larger the TD-error is, the greater the gap between the predicted value of the current sample and the target value will be. This means that this sample is more likely to contain important information, such as it might be a sample that can isolate multiple test points, or a sample located at a critical decision point.

After calculating the TD-error for each sample, a priority function p=f(δ) is used to determine the priority of the samples. The specific approach is to set p=∣δ ∣+ϵ, where ϵ is a small positive number used to ensure that all samples have non-zero priorities. This ensures that even samples with a TD-error of 0 have a certain chance of being selected. In the experience replay buffer, in addition to storing the experience samples $\left(s_{j},a_{j},r_{j},s_{j+1}\right)$, the priority p of each sample also needs to be stored. This can be achieved using a priority queue or an array with priority information.

Next, sampling is carried out based on the priority of the samples. One common sampling method is based on probability distribution, and this probability distribution is related to the priority of the samples. Specifically, the probability formula for selecting sample i is shown as

(19) $$P(i)=\frac{p_{i}^{\alpha }}{\sum_{k}p_{k}^{\alpha }},$$

where P(i) is the priority of sample i; α is the PER prioritization exponent hyperparameter that determines the degree of influence on priority control. When α=0, the sampling becomes uniform sampling; the larger α is, the greater the probability that the samples with higher priority will be selected.

To avoid always selecting the sample with the highest priority, importance sampling weight $w_{i}$ is applied to adjust the contribution of the sampled samples to the training, and its calculation formula is shown as

(20) $$w_{i}=\frac{{\left(N\times P(i)\right)}^{-\beta }}{\underset{j}{\max}w_{j}},$$

where N is the total number of samples in the experience buffer zone; β is the PER importance sampling hyperparameter used to adjust the degree of adjustment of the importance sampling weights.

### 3.3. Algorithm Implementation Steps

**Figure 4:**
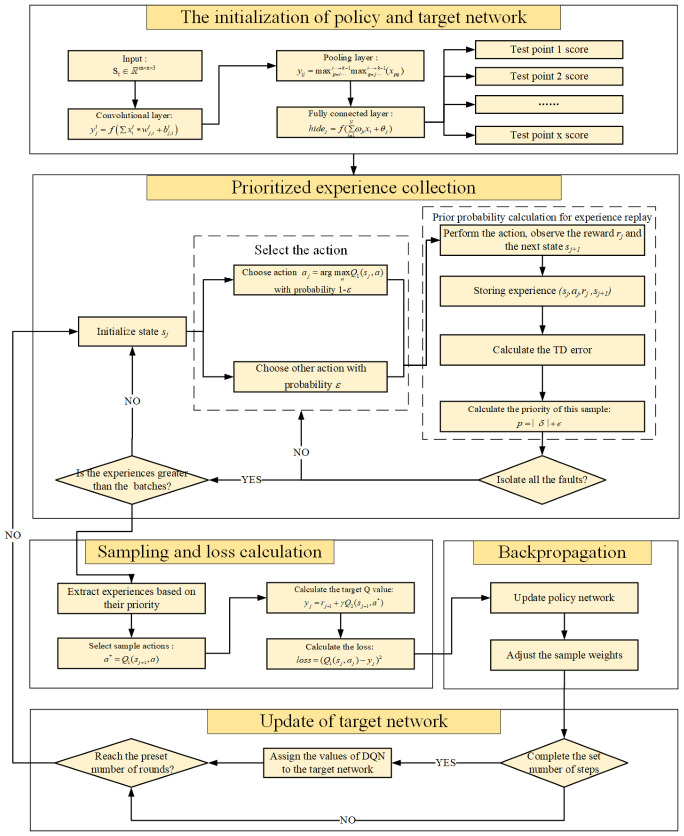
The flowchart of PE-DDQN.

Based on the traditional DQN, DDQN and PER are introduced to construct a PE-DDQN model for testability strategy optimization. The specific implementation of the algorithm process of PE-DDQN is shown in Figure 4. The steps for establishing the PE-DDQN can be summarized as five steps as follows.

(1) The initialization of policy network and target network. Firstly, the parameters of the DQN and the target network need to be initialized separately. The network structure adopts a convolutional neural network.
(2) Priority experience collection. The agent selects test points through a greedy strategy and obtains the next state and the reward for that step from the MVD matrix to obtain experience samples and store it in the buffer pool. This process continues until all faults are isolated and the termination state is reached. At the same time, for each sample, the TD error is calculated, and a priority is assigned.
(3) Sampling and loss calculation. When the number of samples in the pool is greater than the batch size, the algorithm will select samples according to priority. The agent uses the policy network to select actions and calculate the current Q-value, and uses the target network to calculate the target Q-value.
(4) Backpropagation. The policy network parameters are updated through backpropagation, and the updated parameters consist of two parts. One part consists of the parameters of the convolutional neural network in DQN, and the second part is the weight parameters of the PER pool.
(5) Update of the target network. After a certain number of training sessions, the parameters of the DQN will be assigned to the target network for the update of the target network.

After completing the steps, the agent will return to the second step to begin collecting priority experience samples and initiate the next round of training. The algorithm will repeat the above steps until the network converges or the preset number of training iterations is reached.

## 4. Experimental Verification

Before delving into the specific case studies and algorithmic comparisons, the network architectures and overall training configurations are established by adjusting parameters. The detailed structural parameters of the DQN utilized for state feature extraction are summarized in Table 1.

Furthermore, to ensure strict methodological reproducibility, all vital hyperparameters involved in the training phase of the PE-DDQN agent are explicitly defined in Table 2. This set of hyperparameters can enable the algorithm to balance both training stability and training efficiency during the subsequent training process.

### 4.1. Experimental Case 1

#### 4.1.1. MVD-Matrix Modeling

**Figure 5:**
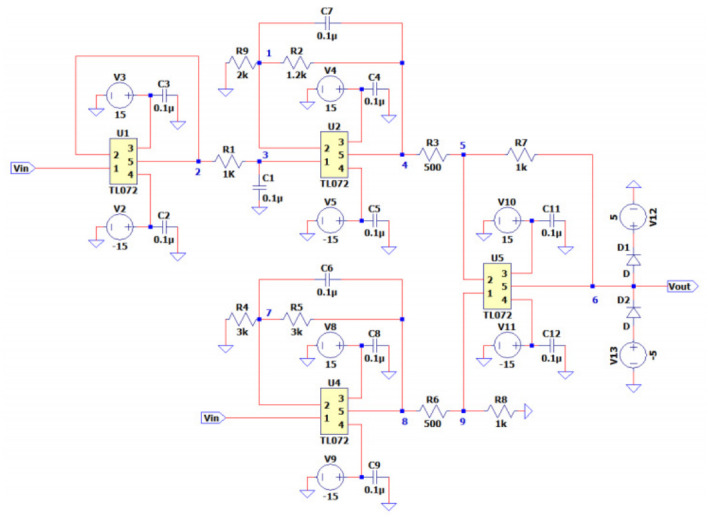
Sampling circuit.

The first research object is a sampling circuit composed of an amplification and subtraction circuit, which is shown in Figure 5. It is an important component of a digital signal system, which can provide a reliable data source for subsequent digital signal processing and enhance the flexibility and multi-band adaptability of the system. A total of 14 fault conditions (open/short circuit of resistors and short circuit of capacitors) are defined, and together with the normal state, there are 15 working conditions, and 9 test points are set.

The circuit voltage excitation is set as a sinusoidal AC voltage source with an amplitude of 4 V and a frequency of 1 kHz. The transient analysis voltage signal is used until stabilization. Based on the actual experiment and exported data, the set simulation time is observed to be 10 ms, and the maximum time step is 1 ms. The experiment recorded the effective values of each test point under different fault conditions. Based on the 0.3 V voltage drop threshold, the MVD matrix of the sampling circuit is constructed by fuzzy integer encoding, as shown in Table 3. The redundant test point T3 is eliminated because its multi-valued fault response column is completely identical to test point T1, offering no additional fault isolation information.

#### 4.1.2. Optimization of Test Sequence Length

For this simulation circuit, the failure rates of all components are approximately equal, and the difficulty of conducting tests at each test point is comparable. Therefore, our objective is to take the fewest number of steps to quickly isolate all faults. With the aim of minimizing the test sequence length, the corresponding reward function is selected. The PE-DDQN testability model is constructed and trained according to the steps described in the third subsection. The maximum number of iterations is 200.

**Figure 6:**
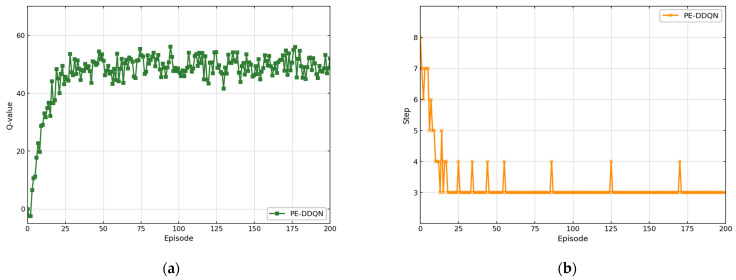
The training process of PE-DDQN: (**a**) average Q-value; (**b**) required steps.

The average Q-value during the training process is shown in Figure 6a. When the average Q-value rises to a certain extent, it enters a state of stable small fluctuations. This indicates that the algorithm has achieved a certain degree of convergence and has found a relatively stable strategy. The small fluctuations are caused by the setting of the exploration rate. However, overall, the average Q-value remains within a relatively stable range, indicating that the algorithm has good stability. This stability enables the agent to make relatively consistent decisions when facing similar situations, improving the reliability of the algorithm in practical applications.

The steps required for the agent to reach the destination are shown in Figure 6b. Initially, the required test sequence length fluctuates considerably due to the agent’s exploration and unfamiliarity with the environment, leading to inefficient actions and longer sequences. As training proceeds, the agent learns and optimizes its strategy, so the sequence length decreases rapidly. After stabilizing at a low level, minor occasional fluctuations demonstrate the algorithm’s adaptability to environmental uncertainties.

Subsequently, the initial state is selected and sent to the trained model. The test point with the highest Q-value is taken and updated to the next state. This step is repeated until all faults are isolated. The final output test sequence is [T5, T6, T2]. According to Table 3, the diagnostic tree can be plotted based on the output test sequence.

**Figure 7:**
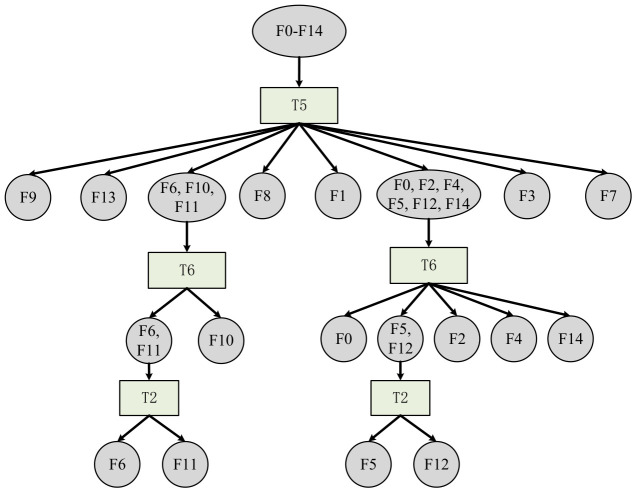
Diagnostic tree based on test sequence length.

At the initial state, all faults are not distinguished, meaning all faults are in a fuzzy group. Firstly, the agent selects test point T5, which divides the fault group into eight parts: {F9}, {F1}, {F8}, {F3}, {F0, F2, F4, F5, F12, F14}, {F7}, {F13}, {F6, F10, F11}. Then, the test point T6 in the current state is selected, which divides the two incomplete isolated fuzzy groups into {F0}, {F2}, {F4}, {F5, F12}, {F14}, {F6, F11}, {F10}. These fuzzy groups, along with the faults F9, F1, F8, F3, F7, F13, isolated in the previous round, indicate that there are currently 11 isolated faults and two incomplete isolated fuzzy groups of faults. Finally, the agent selects test point T2 for the test. At this point, all faults will be completely isolated. According to the above logic, the drawn diagnostic tree is shown in Figure 7. In the figure, the green boxes represent the selected test points, and the gray circles represent the faulty fuzzy groups.

By adjusting the initial seed during the model’s operation, five independent model training sessions are conducted, and the final test sequence is the output. The length output by the five experiments is all three, and the results are shown in Table 4. From the output results, it can be seen that although the test sequence and sorting methods vary each time, they can each successfully isolate every fault through three testability tests. This indicates that there is more than one sequence that can isolate all the faults with the minimum number of diagnostic attempts, but they are all the shortest test sequences.

### 4.2. Experimental Case 2

#### 4.2.1. MVD-Matrix Modeling

**Figure 8:**
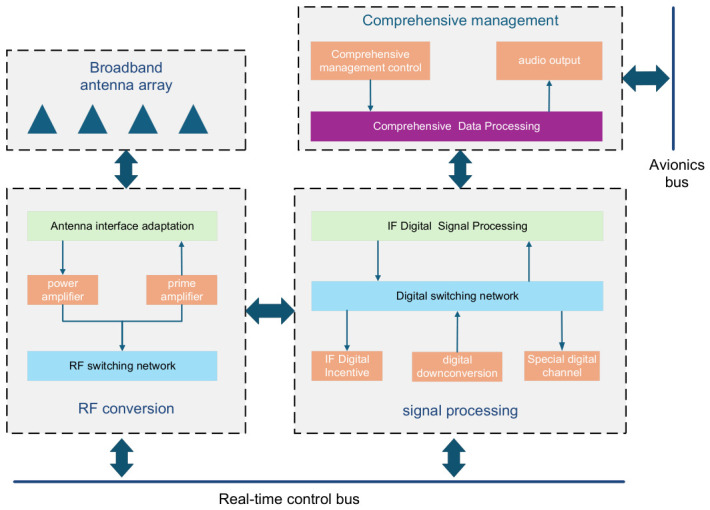
The structure of MIRFS.

The second case study focuses on the MIRFS, which is a shared electronic platform that relies on radio frequency integration, software integration, and aperture integration technologies. Currently, it has been widely applied in military electronic countermeasures and civil aircraft communication. The structure diagram of MIRFS studied in this paper is shown in Figure 8. It replaces the complex discrete antenna apertures with a broadband distributed multi-functional aperture, and realizes resource sharing and function reconstruction by using scheduling control algorithms and software programming. It mainly consists of three core modules: radio frequency (RF) processing, signal processing, and integrated management. These modules work in synergy to implement the functions from RF signal reception and processing to integrated management and output. Meanwhile, it interacts with the avionics bus and is integrated into the overall avionics system.

Based on the system structure composition of the aforementioned MIRFS, the MVD matrix model of MIRFS is shown in Table 5. This fault information covers the normal state of the system and 20 typical faults, and also provides the occurrence probabilities of these faults. The occurrence probabilities are derived based on the operational patterns of the system in actual engineering. The test information contains 10 test points derived from the MIRFS architecture, with their respective execution costs specified. The test items are: T1 (antenna Standing Wave Ratio), T2 (Power amplifier (PA) output power), T3 (RF switch time), T4 (Intermediate frequency (IF) amplitude), T5 (down-conversion gain), T6 (digital channel), T7 (data processing), T8 (control bus load), T9 (avionics bus rate), and T10 (system connectivity). The execution costs are set differently based on the complexity and operational difficulty of the detection modules. The values in the matrix represent the detection response values of each fault at the corresponding test points. Test points with insignificant fault differences will be grouped into the same fuzzy category.

#### 4.2.2. Optimization of Test Cost

**Figure 9:**
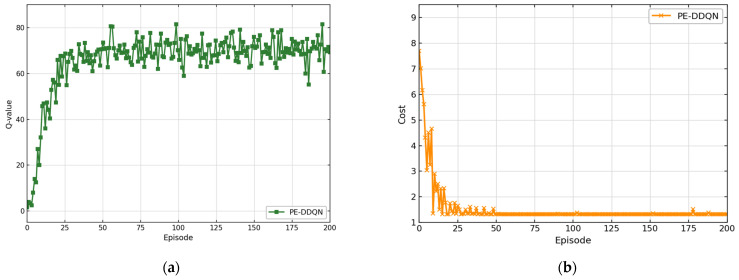
The training process of PE-DDQN: (**a**) average Q-value; (**b**) test cost.

For the MIRFS system, the complexity and operational difficulty of its test points are different, and the failure probabilities of each component also vary. Therefore, we consider using a low testing cost to isolate all the faults. Similarly, with the aim of minimizing the test cost, the corresponding reward function is selected. The PE-DDQN testability model is constructed and trained according to the steps described in the third subsection, whose process is shown in Figure 9. Compared with the reward function based on the test sequence length, the reward function based on the test cost increases the reward for isolating faults, and thus, the overall Q-value during the training process is higher than that of the test sequence length. Meanwhile, the test cost output by the model also tends to converge.

**Figure 10:**
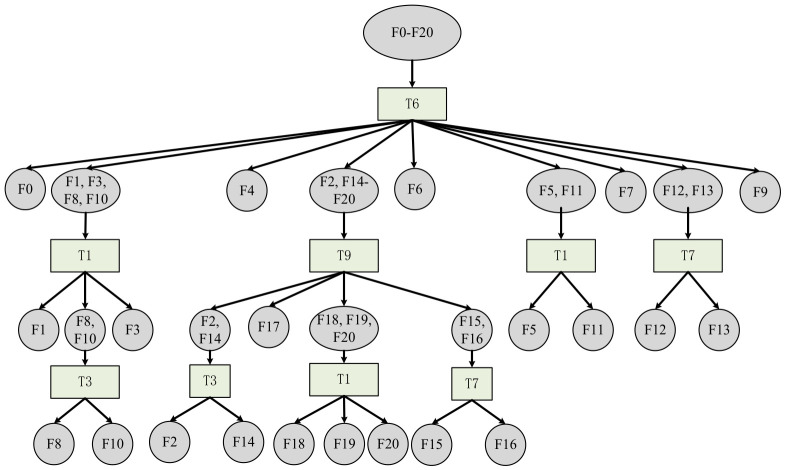
Diagnostic tree based on test cost.

By inputting the initial state vector into the trained model, the test sequence corresponding to the minimum test cost is output. The mean and standard deviation of the results from five independent repeated experiments with random seeds are 1.2404 ± 0.0040. The lowest testing cost among the five experiments is 1.2370, and the costs for each fault are shown in Table 6. The final output of the test point set includes [T6, T1, T9, T7, T3]. Unlike the optimization of test sequence length, this is merely a possible set of measurement points that might be adopted during the testing process, rather than a fixed testing sequence. The actual testing process will dynamically adjust the test points based on the results of each diagnostic step, thereby forming a dynamic diagnostic tree. The execution begins with T6, which serves as a highly discriminative root node, isolating faults F0, F4, F6, F7, F9 and F12, F13 (via further branches). Following the diagnostic tree, the agent dynamically selects subsequent tests based on the specific ambiguity group remaining. For example, for the subgroup [1,3,8,10], the agent performs T1 to isolate F1 and F3, and further utilizes T3 to distinguish F8 and F10. Notably, for the complex subgroup [2,14,15,16,17,18,19,20], the agent adopts T9, followed by T1, T3 and T7 in a branching manner, ensuring that each fault is isolated through the shortest path relative to its individual probability-weighted cost. The diagnostic tree is shown in Figure 10. Enumeration verification confirms that this test yields the lowest test cost.

#### 4.2.3. The Comparison of the Two Reward Mechanisms

In order to compare the characteristics of the two reward mechanisms, the length of the test sequence for MVD matrix of MIRFS is optimized. Under the test sequence length optimization reward function, the output test sequence is T9, T3, T1 and T7, and the total test cost corresponding to the output sequence is 4.948. The comparison of the outputs of the two reward mechanisms is shown in Table 7. The cost differences between the two mainly lie in two aspects. The algorithm based on the test cost optimization reward mechanism considers how to isolate the most likely occurring fault F0 with the smallest test cost combination as soon as possible, while the algorithm based on the test sequence length optimization reward mechanism considers how to isolate all faults more quickly. Although the number of test points has increased, it has, to some extent, reduced the test cost and enabled the test to be conducted in a more economical manner.

### 4.3. Contrastive Analysis

#### 4.3.1. Comparison with Traditional Algorithms

In order to study the performance differences in the proposed algorithm compared to the traditional testability strategy optimization methods, as well as its outstanding superiority in high-dimensional matrix, this paper compares the optimization performance of the proposed PE-DDQN algorithm with that of six traditional optimization algorithms under various dimensional MVD matrix, including ant colony (AC), growing, rollout, info-entropy greedy (IG), genetic algorithm (GA), and particle swarm optimization (PSO). The MVD matrix dimension used in the contrastive experiment increases in dimension, ranging from 20 to 200. For each matrix dimension, 30 repeated experiments are conducted using seven different algorithms, ensuring the reliability of the experimental results. Elements within the matrix are randomly generated, and a certain change is added in each repeated experiment. Since the optimal solution for the shortest sequence length often has multiple possibilities, this optimization objective is relatively simple and cannot fully reflect the superiority or inferiority of the algorithm. Therefore, the complex and representative test cost is selected as the optimization objective.

Table 8 presents the average test cost and standard deviation obtained from 30 experiments of seven algorithms across various MVD matrix dimensions. It can be observed from Table 8 that some heuristic algorithms, such as GA and PSO, maintain a relatively good ability to search for the optimal test cost when the matrix dimension is relatively small. The average value of the results from multiple experiments is not significantly different from that of PE-DDQN. However, as the dimension of the matrix increases, the average test cost of all traditional optimization algorithms gradually exceeds that of PE-DDQN during the process of continuous increase, and thus falls into a local optimum. In contrast, the proposed PE-DDQN algorithm maintains the lowest average test cost across all tested high-dimensional scenarios, demonstrating stable and superior cost optimization capability. This is because these heuristic methods require the analysis and calculation of a large amount of data, involving numerous variables and complex logical relationships. As the scale of the system continues to expand and its complexity deepens, the computational load will show an exponential growth trend, making it impossible to find the optimal solution. Meanwhile, the relatively small standard deviation of the PE-DDQN algorithm as a whole also indicates that its stability is higher compared to other algorithms, and it has broader application value.

In order to further quantitatively verify the statistical reliability of the performance advantages of the PE-DDQN algorithm under various conditions of dimensions, a two-way analysis of variance (ANOVA) is conducted based on dimensions and algorithms. Taking into account that there might be differences in variance among different datasets, the ANOVA with HC3 robust standard errors is employed for the correctional test. The ANOVA analysis results for the main effects of the algorithms are F=11001 and p<0.001 This indicates that, in general, these eight algorithms have fundamental performance differences in terms of overall cost optimization capabilities. The analysis results of the interaction effect are F=586.2 and p<0.001, which reveals a highly significant interaction effect between the algorithm and matrix dimension factors.

**Figure 11:**
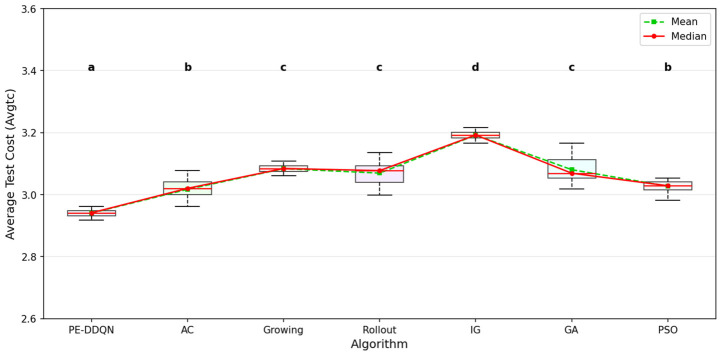
Comparison of test cost for 30 experiments (Dimension = 200).

Subsequently, we conduct a one-way ANOVA and pairwise post hoc test in different dimensions, with the algorithm serving as the variable. Considering that the variances are different among the groups, we employ the Welch’s ANOVA and Games–Howell post hoc tests. In the case of a 200-dimensional matrix, the one-way ANOVA result is F=1018 and p<0.001, demonstrating significant overall differences in optimization performance among all compared algorithms, which justifies carrying out the subsequent experiments. Based on this, an independent pairwise post hoc test is conducted between each algorithm, with corresponding *p*-value calculated to quantify performance differences and statistical significance via a comparison matrix, which is shown in Table 9. The elements in the matrix represent the *p*-value corresponding to the two algorithms. The results show that all *p*-values of PE-DDQN against other algorithms are less than 0.001, indicating that PE-DDQN achieves significantly lower test cost than all comparative algorithms with extremely significant statistical advantages. Among them, the *p*-value corresponding to the multi-valued rollout algorithm is the smallest, meaning that the performance gap between PE-DDQN and the rollout algorithm is the most prominent under high-dimensional conditions. In addition, non-significant pairwise comparisons further show the grouping of significance letters, where algorithms sharing identical letters exhibit no statistically significant performance difference, where p≥0.05. Figure 11 shows the error range of costs in the 30 experiments for each algorithm and the grouping situation of algorithm performance, intuitively reflecting the stability and superiority of the PE-DDQN algorithm in terms of test cost. Based on the statistical significance of the letter markings, the seven algorithms were divided into four distinct groups. Among them, PE-DDQN is classified as group a, indicating its significant advantages and stability compared to other algorithms. AC and PSO are grouped as group b. Growing, GA, and Rollout are grouped as group c. IG is classified as group d alone, which indicates that the performance of this algorithm is the worst.

We also conduct a series of the aforementioned experiments in other matrix dimensions. Among them, except for the PE-DDQN algorithm, the grouping situation of the other algorithms will change, indicating that they will fall into a local optimum as the matrix dimension increases, thereby leading to a decrease in algorithm stability. However, the PE-DDQN algorithm is always classified into group a, which is the group with the best performance, demonstrating the reliability of the proposed method.

**Figure 12:**
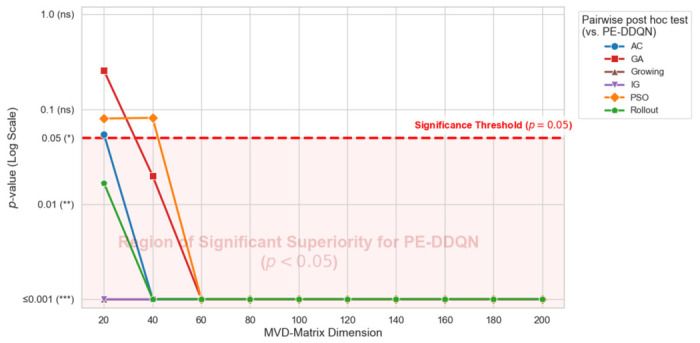
The changes in the *p*-values of each dimension.

The analysis results of the interaction effect show that the performance gap between algorithms is highly dependent on the dimensionality of the problem. To visually unravel this dynamic statistical divergence, Figure 12 illustrates the sequential evolution of the pairwise *p*-value against PE-DDQN across all evaluated dimensions. The vertical axis of the figure shows pairwise comparison *p*-values on a logarithmic scale, and the red dashed line denotes the significance threshold of p=0.05. The area above the red line corresponds to p>0.05, indicating no statistically significant difference between algorithms, which is marked as “ns” (not significant) on the axis ticks. The single asterisk * at p=0.05 represents a significant difference, the double asterisk ** at p=0.01 represents a highly significant difference, and the triple asterisk *** at p≤0.001 represents an extremely significant difference. The red shaded region below the red line satisfies p<0.05, demonstrating that PE-DDQN achieves statistically superior performance compared with other algorithms. As the dimension of the MVD matrix increases, the *p*-values corresponding to most comparison algorithms keep decreasing, which further strengthens the statistical significance of PE-DDQN’s superiority. At the initial stage, when the dimension is 20, advanced heuristics such as GA and PSO establish perfect statistical parity with PE-DDQN, confirming their adequacy for simple state spaces. However, as the combinatorial explosion of the multi-valued matrix intensifies, a collective downward trajectory into the significance domain (p<0.05) is prominently observed. Heuristics like rollout and growing dive below the threshold early at very small dimensions, while GA and PSO manage to sustain equivalence up to 40 and 60 dimensions separately before undergoing a sharp drop. Eventually, at the very high complexity dimension, the *p*-value of all traditional algorithms has uniformly dropped to the lowest limit of 0.001. This dynamic significance evolution rigorously demonstrates that traditional methods suffer from acute performance decay under the curse of dimensionality, whereas PE-DDQN exhibits robust cost-suppression and outstanding architectural scalability for high-dimensional complex systems.

**Figure 13:**
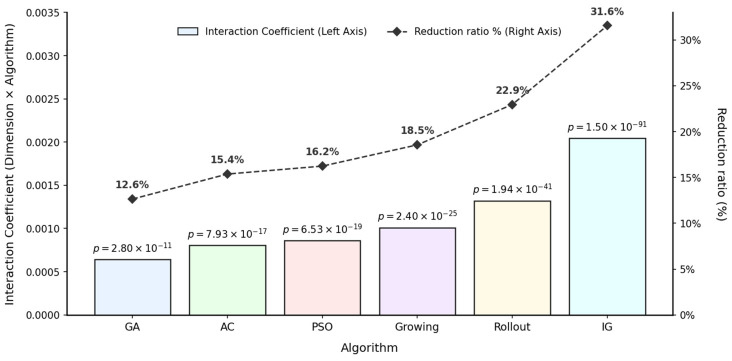
Interaction term coefficient and its significance compared with traditional algorithms.

Table 8 indicates that an increase in the dimension of the MVD matrix will lead to an increase in the test costs output by each algorithm. To compare the differences in the growth of test cost across various algorithms as dimensionality increases, we construct a multiple linear regression model incorporating interaction terms with PE-DDQN serving as the baseline. In this model, the interaction coefficient mathematically represents the exact difference in the fitted “dimension-cost” slopes between a traditional algorithm and PE-DDQN. We also calculate the ratio of each interaction coefficient to the fitted “dimension-cost” slope of the corresponding compared algorithm. This reduction ratio intuitively reflects the degree to which PE-DDQN outperforms traditional algorithms in mitigating the curse of dimensionality. These results are presented graphically using bar charts in Figure 13, and the significant *p*-values of the corresponding interaction terms are also annotated. This confirms that as the complexity of the system increases, the growth rate of testing costs of the traditional algorithm is significantly faster than that of PE-DDQN. Among them, the PSO algorithm shows the fastest degradation rate as the dimension increases. The reduction ratio line also definitively shows that the average increase rate of the test cost output by PE-DDQN decreases by 12.6% to 31.6% compared to various traditional algorithms as the dimension of the MVD matrix increases, which proves that the PE-DDQN possesses a statistically superior capability to suppress cost growth in high-dimensional environments, outperforming every single baseline heuristic algorithm.

To further evaluate the computational complexity and scalability of the proposed framework, Table 10 presents the average test time of different algorithms across varying MVD matrix dimensions from 20 to 200 (unit: seconds). All experiments in this study are conducted on a high-performance mobile workstation equipped with an Intel Core i7-11800H CPU @ 2.30 GHz (8 cores), 32 GB of DDR4 RAM (3200 MHz), and an NVIDIA GeForce RTX 3060 Laptop GPU. The proposed PE-DDQN framework and all comparative algorithms are implemented with CUDA enabled for hardware acceleration. As shown in Table 10, simple heuristic algorithms such as Growing and IG maintain extremely low computational times across all dimensions due to their greedy nature. However, as demonstrated in previous sections, this speed comes at the severe cost of falling into local optima and yielding poor diagnostic strategies. Conversely, complex heuristic algorithms like AC suffer from a severe combinatorial explosion, with test times surging exponentially to 457.13 as the dimension scales to 200.

It is noteworthy that population-based heuristics like GA and PSO maintain moderate computational times, which are lower than that of PE-DDQN. However, the optimization performance of these two common heuristic algorithms significantly deteriorates in high-dimensional scenarios, and their speed advantage is unable to compensate for the deficiency in their optimization performance.

Since PE-DDQN employs deep neural networks for fitting, its running time is longer compared to ordinary heuristic algorithms, but it remains within a controllable range overall. It effectively avoids the exponential time explosion seen in AC while dedicating a reasonable and controllable amount of computational time to successfully overcome the curse of dimensionality and find globally optimal strategies. Consequently, PE-DDQN offers the best trade-off between computational scalability and optimization accuracy for complex systems.

Overall, traditional heuristic optimization algorithms are prone to performance degradation and local optimum trapping when facing high-dimensional MVD matrix optimization problems. Benefiting from deep reinforcement learning-driven feature extraction and adaptive decision-making, the proposed PE-DDQN algorithm can effectively cope with the dimensionality curse. It achieves better test cost optimization performance while ensuring acceptable computational efficiency, which is more suitable for high-dimensional engineering application scenarios.

#### 4.3.2. Ablation Experiment

**Figure 14:**
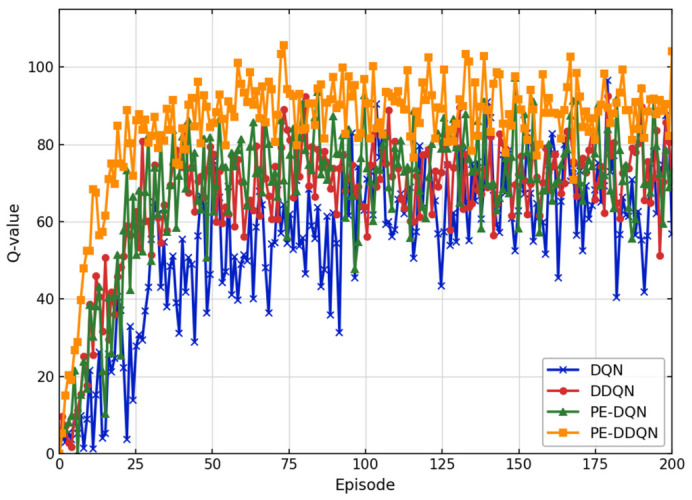
The average Q-value of the four algorithms.

In order to highlight the efficiency and stability of the convergence of PE-DDQN during the training process, DQN, DDQN, PE-DQN and PE-DDQN are respectively used to optimize the test points of a random high-dimensional MVD matrix, and the cost optimization objective is adopted. The average step-wise Q-values of the four algorithms during training are illustrated in Figure 14.

As shown in Figure 14, DQN exhibits severe Q-value overestimation bias, which causes the agent to overprioritize actions with artificially inflated Q-values while neglecting potentially optimal action spaces. Consequently, it fails to explore high-reward action strategies, leading to low cumulative rewards, a globally suboptimal policy, and persistently low converged Q-values. By decoupling action selection from value evaluation, DDQN effectively alleviates overestimation and achieves a higher steady-state Q-value with reduced training fluctuations compared with DQN, yet its convergence speed remains relatively limited. Benefiting from the policy-enhanced module, PE-DQN achieves the fastest convergence rate by rapidly extracting critical optimization information from early training samples, though its training stability is inferior to DDQN.

Further integrating the advantages of policy enhancement and double-network structure, PE-DDQN not only inherits the rapid convergence characteristic of PE-DQN but also retains the low-fluctuation merit of DDQN. Its prioritized experience replay mechanism further accelerates the learning of high-impact key samples containing optimal action information. It can avoid wasting computational resources on low-value samples and guide more efficient policy optimization toward the global optimum. Overall, PE-DDQN achieves the best comprehensive performance in terms of convergence speed, training stability, and final Q-value level among all compared algorithms.

To verify the enhancement effect of the DDQN and PER module introduced in this paper on the testability strategy optimization, as well as the gain in adaptability of the model to a high-dimensional MVD matrix, we conduct test cost optimization experiments for the aforementioned ablation models. These experiments are under different MVD matrix dimensions with randomly generated matrix elements. For each matrix dimension ranging from 20 to 200, 30 independent repeated experiments are implemented for the four algorithms. Table 11 summarizes the average test cost and standard deviation.

**Figure 15:**
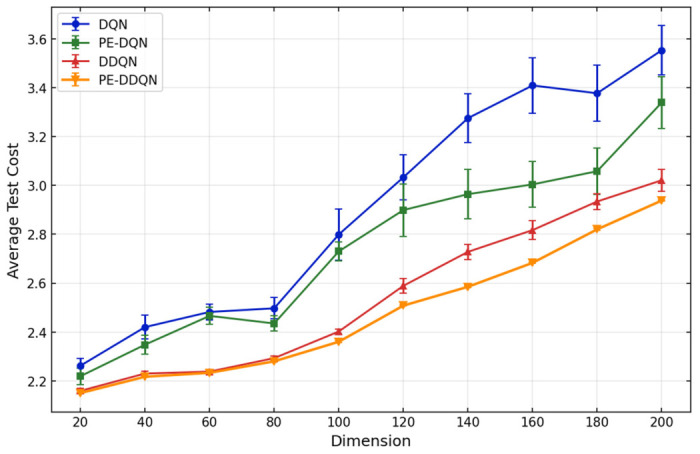
Comparison of average test cost of four algorithms under different dimensions.

In terms of optimization performance, DQN and PE-DQN suffer from severe overestimation bias due to the absence of a double-network mechanism, which traps the models in suboptimal local minima and prevents them from obtaining globally optimal test strategies. Correspondingly, their larger standard error range in Figure 15 indicates poor stability and significant random fluctuations across repeated runs. By introducing the double-network structure, DDQN effectively mitigates overestimation and performs well in low-dimensional scenarios. Nevertheless, as the dimension increases, the diversity and redundancy of experience samples will grow considerably. Without prioritized sampling, DDQN exhibits degraded optimization performance and moderate stability in high-dimensional cases. By integrating the double-network structure and prioritized experience replay, PE-DDQN achieves the lowest average test cost across all dimensions, accompanied by the narrowest standard error bars in Figure 15, which verifies its superior optimization accuracy and robustness.

To verify the advantages of PER and DDQN mechanisms in high-dimensional environments, a two-way ANOVA is performed on the test cost of each algorithm. The ANOVA analysis results for the main effects of the algorithms are F=140.8 and p<0.001. This indicates that there are significant differences among the algorithms as a whole. The analysis results of the interaction effect are F=7.060 and p<0.001, which reveals an extremely significant interaction effect between algorithm type and environmental dimension. Combined with Figure 15, it is the PE-DDQN model that outperforms the other three ablation models, and the advantage becomes more pronounced as the dimension increases.

**Figure 16:**
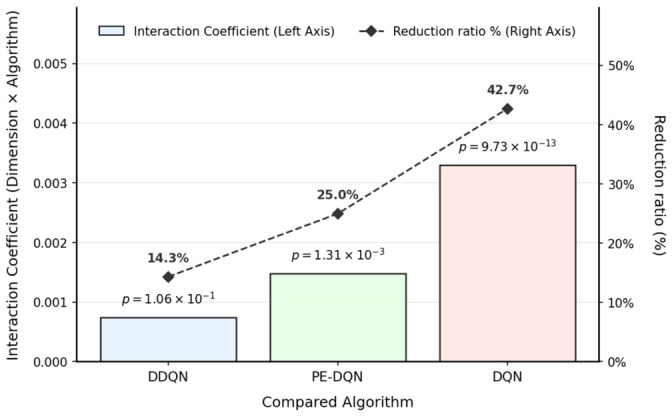
Interaction term coefficient and its significance compared with the ablation model.

Based on several ablation algorithms, we also construct a multivariate linear regression model with interaction terms. Taking the proposed PE-DDQN as the baseline, the interaction coefficients between it and each ablation model are calculated, as well as the reduction ratio of each interaction coefficient to the fitted “dimension-cost” slope of the corresponding ablation model, which is shown in Figure 16. The calculation results clearly demonstrate that the introduction of these two mechanisms indeed leads to a significant improvement in performance. DQN suffers the steepest performance degradation with growing dimensions without PER or DDQN constraints. The PE-DQN achieves a notably lower interaction coefficient by easing high-dimensional feature sparsity-induced learning bottlenecks via prioritized sampling. The DDQN separates action selection from value estimation. Its interaction coefficient is already relatively low, and there is no significant difference from PE-DDQN. However, Figure 15 shows that the introduction of PER to it can still reduce the test cost in a high-dimensional matrix environment. The reduction ratio line also definitively shows that the average increase rate of the test costs output by the PE-DDQN algorithm is approximately 42.7% lower than DQN when the dimension of the MVD matrix increases, and it is also lower than the other two ablation models.

From the above experiments, it can be seen that the introduction of PER and DDQN has a significant effect in high-dimensional environments. DQN typically struggles with severe overestimation bias and sample redundancy. The introduction of DDQN addresses the overestimation issue by decoupling action selection from value evaluation. This stabilization is crucial in large-scale testability environments, as it prevents the agent from overvaluing suboptimal test points and getting trapped in local minima during complex fault isolation sequences. On the other hand, the PER mechanism specifically tackles the combinatorial explosion of experience samples. By prioritizing the selection of high-value transfer states, PER ensures that the network does not waste computing resources on redundant or information-less test steps. Consequently, while DDQN provides a stable and accurate evaluation baseline, PER accelerates convergence and enhances the discovery of globally optimal strategies. Their synergistic integration into the PE-DDQN framework forms a highly persuasive and robust solution for large-scale testability strategy optimization.

In summary, the comprehensive experimental evaluations conducted in this section rigorously demonstrate the superiority of the proposed PE-DDQN framework from multiple perspectives. First, extensive comparative experiments are performed against six representative heuristic algorithms across a wide range of MVD matrix dimensions ranging from 20 to 200. Exhaustive statistical significance analyses, including ANOVA and pairwise post hoc tests, provide robust evidence that PE-DDQN achieves significantly lower testing costs (p<0.001). Furthermore, multiple linear regression analyses incorporating interaction terms mathematically confirmed that PE-DDQN possesses a statistically superior capability to suppress the cost-escalating effect induced by the curse of dimensionality. The execution times of different algorithms also confirm that the proposed algorithm maintains an overall controllable computational complexity. It indicates that even in high-dimensional scenarios, the proposed PE-DDQN can derive a superior diagnostic strategy within an acceptable execution time. Finally, comprehensive ablation studies are conducted to explicitly isolate and quantify the individual contributions of the DDQN and PER mechanisms. The results rigorously proved their synergistic necessity in preventing overestimation bias and overcoming sample redundancy. Together, these thorough theoretical and empirical validations confirm that PE-DDQN offers a statistically superior, highly scalable, and structurally robust solution for complex system diagnostics.

## 5. Conclusions

This study proposed a novel testability strategy optimization method to address the issue of poor performance of the optimization strategy based on a high-dimensional MVD matrix. The proposed method is applied to the sampling circuit and the MIRFS, and a high-dimensional matrix simulation experiment is conducted to compare it with other algorithms. The main conclusions are as follows:

(1) The Markov decision model in terms of test sequence length and test cost is effective. By linking testability strategy optimization with the Markov decision process, the optimization model is established. The sets of elements and reward functions for test sequence length and test cost optimization are established. The reward mechanisms enable the intelligent agent to effectively minimize the test sequence length or test cost while isolating all faults.
(2) The construction of the DQN enables the agent to fit the Q-value of test points and select the optimal test point. A deep neural network is used to approximate the Q-value functions, and the selection of the testability strategy will be based on this neural network. Experience samples are obtained through the MVD matrix, and based on the Markov decision model, the parameters of the Q-network are updated to fit the Q-value through sample learning. The establishment of the DQN ensures that the model can select the optimal test point in each fault isolation status.
(3) The introduced DDQN and PER mechanism can effectively enhance the model’s adaptability to the high-dimensional matrix environment. The optimization algorithm based on PE-DDQN can effectively alleviate the overestimation problem of DQN and enable the model to focus on important samples, thereby improving the convergence. Thus, it significantly improves the training efficiency of the model and the reliability of the output testability strategy when dealing with a high-dimensional MVD matrix.

In summary, the overarching contribution of this study is the development of a comprehensive DRL-based framework structurally tailored for testability strategy optimization under multi-valued dependency conditions. By strategically adapting and integrating mature reinforcement learning mechanisms into the domain of testability modeling, this research successfully translates advanced artificial intelligence algorithms into practical engineering solutions. The proposed framework effectively addresses the curse of dimensionality and local optimum trapping inherent in traditional methods, providing a robust, highly efficient, and scalable diagnostic tool for modern complex systems.

The future work will focus on establishing an adaptive mechanism for adjusting the reward functions. The weights and parameters of the reward functions will be dynamically changed based on the real-time status of the system and historical performance. This enables the algorithm to better adapt to the dynamic changes in the system, thereby enhancing the effectiveness and adaptability of the reinforcement learning algorithm during its long-term operation.

## Figures and Tables

**Table 1:** The structure of DQN.

Layer Type	Size	Stride	Number of Convolutional Kernels	Explanation
Input layer	m × n × 3	--	--	Receives the 3-channel 2D state tensor.
Convolutional layer	3 × 3	1 × 1	32	Extracts initial coupling features.
Pooling layer	2 × 2	2 × 2	--	Reduces dimensions, retains salient features.
Convolutional layer	3 × 3	1 × 1	64	Expands receptive field for higher-order features.
Pooling layer	2 × 2	2 × 2	--	Compresses spatial redundancy.
Flatten layer	--	--	--	Flattens 2D maps into 1D.
Fully connected layer	512	--	--	Fuses all features globally.
Fully connected layer	n	--	--	Outputs Q-values for n test points.

**Table 2:** Hyperparameters for training.

Parameter	Symbol	Value	Description
Learning rate	lr	0.001	Step size for Adam optimizer
Discount factor	γ	0.99	Importance of future rewards
Replay buffer size	-	20,000	Max experience capacity
Mini-batch size	-	64	Samples per network update
PER prioritization exponent	α	0.6	Degree of prioritized sampling
PER importance sampling	β	0.4~1.0	Importance sampling weight
Target-update interval	-	500 steps	Target network sync frequency

**Table 3:** MVD matrix of sampling circuit.

FT	T1	T2	T4	T5	T6	T7	T8	T9
F0 (normal)	1	0	2	4	4	1	1	2
F1 (R1 open)	0	1	0	1	8	1	1	2
F2 (R1 short)	2	1	3	4	0	1	1	2
F3 (R2 open)	1	0	4	3	6	0	1	2
F4 (R2 short)	1	1	1	4	3	1	1	2
F5 (R3 open)	1	1	2	4	2	1	1	2
F6 (R3 short)	1	1	2	7	9	1	1	2
F7 (R5 open)	1	0	2	5	5	1	1	2
F8 (R5 short)	1	0	2	2	6	1	0	1
F9 (R6 open)	1	0	2	0	8	1	1	0
F10 (R6 short)	1	1	2	7	7	1	1	3
F11 (R7 open)	1	0	2	7	9	1	1	2
F12 (R7 short)	1	0	2	4	2	1	1	2
F13 (C6 short)	1	1	2	6	8	1	2	3
F14 (C7 short)	1	1	3	4	1	1	1	2

**Table 4:** The sequences output from the five experiments.

Number of Experiments	1	2	3	4	5
Output sequence	T5→T6→T2	T6→T2→T5	T6→T2→T5	T6→T2→T9	T9→T6→T2

**Table 5:** MVD matrix of MIRFS.

Fault (Probability)	Fault Message	T1 (1.0)	T2 (1.5)	T3 (2.0)	T4 (1.8)	T5 (2.0)	T6 (1.0)	T7 (1.6)	T8 (0.8)	T9 (1.1)	T10 (2.6)
F0 (0.8500)	No fault	1	1	1	1	1	1	1	1	1	1
F1 (0.0050)	Antenna short circuit	0	0	0	0	0	0	0	1	1	2
F2 (0.0090)	RF switch jamming	3	2	4	3	4	4	1	1	0	0
F3 (0.0050)	Main PA gain loss	1	1	0	0	0	0	0	1	1	2
F4 (0.0080)	Power PA overload	2	3	6	5	6	6	1	1	2	0
F5 (0.0120)	Antenna impedance mismatch	1	1	1	1	1	2	1	1	5	0
F6 (0.0070)	IF signal failure	0	0	3	2	3	3	1	1	8	0
F7 (0.0080)	Conversion gain drift	3	2	5	4	5	5	1	1	4	0
F8 (0.0060)	RF switch input fault	3	2	0	0	0	0	1	1	7	0
F9 (0.0090)	Digital channel overflow	3	2	6	6	7	7	1	1	6	0
F10 (0.0060)	Signal sampling fault	3	2	2	0	0	0	1	1	7	0
F11 (0.0070)	Switch cross fault	3	2	3	3	4	2	1	1	4	0
F12 (0.0080)	IF filter abnormality	3	2	3	2	0	8	5	1	8	0
F13 (0.0090)	Data processing loss	3	2	3	2	2	8	4	1	8	0
F14 (0.0070)	Management command delay	3	2	3	3	4	4	1	1	0	0
F15 (0.0050)	Audio distortion	3	2	3	3	4	4	2	1	8	0
F16 (0.0080)	Switch output fault	3	2	3	3	4	4	3	1	8	0
F17 (0.0050)	IF sampling error	3	2	3	3	4	4	2	2	3	0
F18 (0.0080)	Management comm. interrupt	3	2	3	3	4	4	0	0	1	0
F19 (0.0050)	Control bus error	1	3	2	3	3	4	0	2	1	0
F20 (0.0130)	Bus communication fault	2	2	3	4	2	4	1	0	1	0

**Table 6:** Test cost table for each fault.

No.	Test Points	Cost	No.	Test Points	Cost	No.	Test Points	Cost
F0	T6	0.85000	F7	T6	0.00800	F14	T6→T9→T3	0.02870
F1	T6→T1	0.01000	F8	T6→T1→T3	0.02400	F15	T6→T9→T7	0.01850
F2	T6→T9→T3	0.03690	F9	T6	0.00900	F16	T6→T9→T7	0.02960
F3	T6→T1	0.01000	F10	T6→T1→T3	0.02400	F17	T6→T9	0.01050
F4	T6	0.00800	F11	T6→T1	0.01400	F18	T6→T9→T1	0.02480
F5	T6→T1	0.02400	F12	T6→T7	0.02080	F19	T6→T9→T1	0.01550
F6	T6	0.00700	F13	T6→T7	0.02340	F20	T6→T9→T1	0.04030

**Table 7:** Comparison of two reward mechanisms.

Reward Mechanism	Test Sequence (Set)	Sequence Length	Test Cost	Core Difference
Test sequence length	T9→T3→T1→T7	4	4.948	Fewer steps and faster speed
Test cost	[T6, T1, T9, T7, T3]	5	1.2370	More economical

**Table 8:** Comparison of average test cost and standard deviation for 30 trials.

Dim	PE-DDQN	AC	Growing	Rollout	IG	GA	PSO
20	2.155 ± 0.0029	2.165 ± 0.0017	2.184 ± 0.0020	2.167 ± 0.0021	2.213 ± 0.0032	2.163 ± 0.0020	2.165 ± 0.0018
40	2.215 ± 0.0033	2.235 ± 0.0023	2.275 ± 0.0024	2.242 ± 0.0028	2.246 ± 0.0016	2.229 ± 0.0024	2.226 ± 0.0020
60	2.236 ± 0.0032	2.329 ± 0.0021	2.395 ± 0.0029	2.298 ± 0.0036	2.444 ± 0.0026	2.260 ± 0.0030	2.273 ± 0.0026
80	2.285 ± 0.0024	2.316 ± 0.0043	2.449 ± 0.0025	2.388 ± 0.0034	2.516 ± 0.0022	2.323 ± 0.0025	2.303 ± 0.0033
100	2.360 ± 0.0032	2.426 ± 0.0033	2.554 ± 0.0027	2.474 ± 0.0048	2.634 ± 0.0041	2.457 ± 0.0035	2.445 ± 0.0029
120	2.507 ± 0.0024	2.685 ± 0.0028	2.853 ± 0.0027	2.606 ± 0.0062	2.973 ± 0.0021	2.628 ± 0.0036	2.627 ± 0.0035
140	2.590 ± 0.0026	2.798 ± 0.0050	3.014 ± 0.0022	2.785 ± 0.0058	3.158 ± 0.0032	2.769 ± 0.0061	2.732 ± 0.0038
160	2.683 ± 0.0021	2.887 ± 0.0045	3.061 ± 0.0028	2.886 ± 0.0052	3.177 ± 0.0020	2.870 ± 0.0049	2.818 ± 0.0050
180	2.824 ± 0.0029	2.967 ± 0.0046	3.070 ± 0.0021	2.968 ± 0.0048	3.191 ± 0.0045	2.964 ± 0.0039	2.911 ± 0.0072
200	2.941 ± 0.0022	3.017 ± 0.0055	3.084 ± 0.0023	3.069 ± 0.0059	3.192 ± 0.0024	3.081 ± 0.0078	3.027 ± 0.0031

**Table 9:** Pairwise *p*-value matrix (Dimension = 200).

Algorithm	PE-DDQN	AC	Growing	Rollout	IG	GA	PSO
PE-DDQN	—	<0.001	<0.001	<0.001	<0.001	<0.001	<0.001
AC	<0.001	—	<0.001	<0.001	<0.001	<0.001	0.7114
Growing	<0.001	<0.001	—	0.2892	<0.001	0.9996	<0.001
Rollout	<0.001	<0.001	0.2892	—	<0.001	0.9148	<0.001
IG	<0.001	<0.001	<0.001	<0.001	—	<0.001	<0.001
GA	<0.001	<0.001	0.9996	0.9148	<0.001	—	<0.001
PSO	<0.001	0.7114	<0.001	<0.001	<0.001	<0.001	—

**Table 10:** Comparison of average test time of different algorithms.

Dim	PE-DDQN	AC	Growing	Rollout	IG	GA	PSO
20	8.85	0.18	0.01	0.02	0.00	4.33	2.22
40	11.71	1.64	0.01	0.21	0.01	5.72	3.45
60	17.38	7.32	0.02	0.97	0.02	11.21	6.86
80	21.11	20.96	0.04	2.49	0.04	16.96	11.99
100	38.68	43.41	0.07	9.08	0.10	25.58	16.35
120	44.71	78.36	0.16	20.13	0.15	32.84	25.60
140	53.74	124.00	0.16	24.17	0.20	35.43	28.72
160	65.86	189.22	0.19	33.37	0.20	42.95	38.55
180	79.46	271.93	0.30	50.43	0.29	44.40	39.32
200	98.87	457.13	0.39	69.91	0.37	55.05	49.68

**Table 11:** Comparison of average test time and standard deviation for 30 trials.

Dim	DQN	PE-DQN	DDQN	PE-DDQN
2.155 ± 0.0029	2.262 ± 0.0292	2.219 ± 0.0339	2.159 ± 0.0095	2.155 ± 0.0029
2.215 ± 0.0033	2.420 ± 0.0488	2.348 ± 0.0381	2.230 ± 0.0100	2.215 ± 0.0033
2.236 ± 0.0032	2.482 ± 0.0327	2.466 ± 0.0352	2.238 ± 0.0083	2.236 ± 0.0032
2.285 ± 0.0024	2.497 ± 0.0434	2.435 ± 0.0316	2.293 ± 0.0088	2.285 ± 0.0024
2.360 ± 0.0032	2.799 ± 0.1051	2.730 ± 0.0383	2.402 ± 0.0090	2.360 ± 0.0032
2.507 ± 0.0024	3.033 ± 0.0920	2.899 ± 0.1076	2.589 ± 0.0299	2.507 ± 0.0024
2.590 ± 0.0026	3.275 ± 0.0987	2.964 ± 0.1003	2.728 ± 0.0309	2.590 ± 0.0026
2.683 ± 0.0021	3.409 ± 0.1133	3.004 ± 0.0942	2.817 ± 0.0383	2.683 ± 0.0021
2.824 ± 0.0029	3.377 ± 0.1145	3.058 ± 0.0960	2.934 ± 0.0324	2.824 ± 0.0029
2.941 ± 0.0022	3.553 ± 0.1010	3.339 ± 0.1059	3.021 ± 0.0452	2.941 ± 0.0022

## Data Availability

The data that has been used is confidential.

## Abbreviations

The following abbreviations are used in this manuscript:

D-matrix	Dependency matrix
MVD matrix	Multi-valued dependency matrix
POMDP	Partially observable Markov decision process
DRL	Deep reinforcement learning
DQN	Deep Q-Network
DDQN	Double DQN
PER	Prioritized experience replay
PE-DDQN	Prioritized Experience Double Deep Q-Network
TD-error	Temporal difference error
MIRFS	Multi-functional integrated radio frequency system
RF	Radio frequency
IF	Intermediate frequency
PA	Power amplifier
AC	Ant colony
IG	Info-entropy greedy
GA	Genetic algorithm
PSO	Particle swarm optimization
ANOVA	Analysis of variance
